# DYRK1B Inhibition by AZ191 Sensitizes High-Grade Serous Ovarian Cancer to Niraparib Through Promoting Apoptosis and Ferroptosis

**DOI:** 10.3390/biomedicines14040939

**Published:** 2026-04-20

**Authors:** Yu Gao, Yuanyuan Cao, Junyao Liu, Fang Tong, Xianlin Liu, Jiahui Wang, Peixuan Liu, Yanting Xu, Lu Feng, Pengxin Zhang, Jingchun Gao, Jiwei Liu

**Affiliations:** 1 Department: of Oncology, First Affiliated Hospital, Dalian Medical University, Dalian 116011, China; gaofeather@126.com; 2 Department of Obstetrics & Gynecology, First Affiliated Hospital, Dalian Medical University, Dalian 116011, China; caoyy@dmu.edu.cn (Y.C.); liujunyao0923@163.com (J.L.); tong163fang@163.com (F.T.); xianlinliu2023@hotmail.com (X.L.); wangjiahui_dy@163.com (J.W.); lpx001104@163.com (P.L.); xuyanting0631@163.com (Y.X.); 3 Department of Pathology, First Affiliated Hospital, Dalian Medical University, Dalian 116011, China; fenglusd@163.com (L.F.); zhangpengxin1983@163.com (P.Z.)

**Keywords:** ovarian cancer, DYRK1B, apoptosis, ferroptosis, PARP inhibitor

## Abstract

**Background/Objectives:** The clinical challenges of PARP inhibitors in ovarian cancer include the lack of effective maintenance regimens for homologous recombination proficiency (HRP) patients and the emergence of acquired resistance in initially responsive homologous recombination deficiency (HRD) patients. This study aims to explore the synergistic effect and molecular mechanism of the bispecific tyrosine phosphorylation-regulated kinase 1B (DYRK1B) inhibitor AZ191 combined with the PARP inhibitor Niraparib on high-grade serous ovarian cancer (HGSOC). **Methods:** This study first explored the expression and prognostic significance of DYRK1B in ovarian cancer through bioinformatics analysis. Subsequently, the therapeutic effect of the DYRK1B inhibitor AZ191 combined with Niraparib on HGSOC cells and organoids was evaluated by MTT examination. Flow cytometry and Western blot were used to investigate the synergistic mechanism between the two agents. **Results:** Bioinformatics analysis shows that the high expression of *DYRK1B* in serous ovarian cancer is associated with poor prognosis of the patients. The experiments in vitro have shown that the DYRK1B inhibitor AZ191 can enhance the therapeutic effect of Niraparib on HGSOC cells and organoids, whether HRD-positive or not. Mechanistic studies have shown that the combination of AZ191 and Niraparib can synergistically increase the accumulation of DNA damage, thereby intensifying the apoptosis of HGSOC cells. In addition, the combination therapy induces ferroptosis by inhibiting the Nrf2/SLC7A11/GPX4 axis, thereby exerting cytotoxic effects. **Conclusions:** Our results uncover a novel mechanism by which inhibiting DYRK1B enhances the anti-HGSOC efficacy of Niraparib and may offer a promising treatment strategy to improve the maintenance therapy in both HRD and HRP ovarian cancer patients.

## 1. Introduction

Ovarian cancer, as the most lethal gynecological malignant tumor worldwide, has nearly 324,000 new cases and about 207,000 deaths annually [1]. High-grade serous ovarian cancer (HGSOC) is the most common and aggressive histological subtype, characterized by high genomic instability and frequent TP53 mutations, as well as approximately 50% of patients with homologous recombination repair deficiency (HRD) [2,3]. The introduction of poly (ADP-ribose) polymerase (PARP) inhibitors has revolutionized the therapeutic landscape by exploiting the “synthetic lethality” mechanism in HRD-positive tumors, establishing the current comprehensive management paradigm of “Surgery followed by Chemotherapy then Maintenance Therapy” [4,5].

In recent years, PARP inhibitors have proved highly effective for ovarian cancer patients with DNA double-strand break (DSB) repair defects [6], but their efficacy is limited in those with homologous recombination proficiency (HRP) [7]. Surprisingly, in the key clinical trials evaluating first-line maintenance treatment with the PARP inhibitor Niraparib for newly diagnosed advanced ovarian cancer patients, both PRIME [8] in Chinese patients and PRIMA [9] in European and American populations reported progression-free survival (PFS) benefits in the overall population, regardless of the biomarker status. This benefit increases the proportion of platinum-sensitive relapsed patients, and thereby creates conditions for the subsequent use of platinum-based chemotherapy [6]. Moreover, some patients who respond positively to the initial treatment with PARP inhibitors eventually develop acquired chemotherapy resistance [10]. Thus, how to reverse PARP inhibitor resistance during the first-line maintenance treatment in ovarian cancer while expanding the indications and efficacy of its clinical application remains an urgent challenge.

The dual-specificity tyrosine phosphorylation-regulated kinase (DYRK) family comprises protein kinases that phosphorylate tyrosine and serine/threonine residues, with Class I members (DYRK1A and DYRK1B) and Class II members (DYRK2, DYRK3, and DYRK4) playing diverse roles in cancer development and therapeutic resistance [11]. Studies have shown that DYRK1B, also known as Mirk, was first identified in human testicular tissue and was either absent or expressed at low levels in normal tissues other than the muscle and brain, but was overexpressed in multi-organ solid tumors and cancer cell lines, including ovarian cancer [12]. When *DYRK1B* is “exhausted” or inhibited by drugs or siRNA, it can damage cell survival and induce apoptosis, which is associated with elevated reactive oxygen species (ROS) levels and DNA damage [13,14,15]. At the same time, studies have also found that knockdown of *DYRK1B* induces a transition from the quiescent G0 “dormant” phase with “cancer stem cell” characteristics into the active G1-S-G2/M proliferative cycle, thereby enhancing sensitivity to chemotherapeutic agents. Importantly, DYRK1B depletion does not cause significant cytotoxicity in normal human cells, indicating DYRK1B as a promising therapeutic target for precision cancer therapy [16,17]. AZ191 [18], a 6-azaindole compound identified through systematic protein kinase drug screening, exhibits potent and highly selective inhibitory activity against DYRK1B compared to other family members such as DYRK1A and DYRK2. The above suggests that AZ191 has the potential to be a targeted drug for the treatment of multiple organ malignancies in humans in the future.

Latest research reveals that PARP inhibitor-resistant tumors are highly dependent on the nuclear autoantigenic sperm protein (NASP)-PARP1 axis to maintain histone supply, which supports their elevated DNA replication rates and promotes cell survival [19]. Meanwhile, DYRK1B contributes to transcriptional inhibition following DNA damage by the phosphorylating euchromatichistone-lysineN-methyltransferase2 (EHMT2), thereby coordinating DSB repair [14]. Both are involved in the transcriptional regulation and the maintenance of histone homeostasis during DNA damage repair. Whether targeted inhibition of DYRK1B can mediate the DSB repair barrier to improve the efficacy of PARP inhibitors and overcome chemotherapy resistance deserves further experimental exploration.

In addition to inhibiting DNA damage repair, PARP inhibitors have been reported to induce ferroptosis in ovarian cancer cells by upregulating the expression of CD36 and subsequent induction of excessive fatty acid intake [20]. Previous studies have also found that DYRK1B upregulates the expression of multiple antioxidant genes, such as those encoding superoxide dismutases SOD2 and SOD3, to enhance the tolerance of cancer cells to high ROS levels [12,13,17,21]. ROS plays a key role in initiating lipid peroxidation and ferroptosis, which significantly influences chemotherapy resistance [22]. Therefore, targeting DYRK1B represents a promising novel clinical strategy for increasing PARP inhibitor sensitivity.

This study initially employed bioinformatics approaches to investigate the biological functions of DYRK1B in ovarian cancer progression and its regulatory role in tumor immune infiltration. We then investigated the synergistic effects and underlying mechanisms of combined treatment with the DYRK1B inhibitor AZ191 and the PARP inhibitor Niraparib in HGSOC cell lines and organoids. We aimed to explore whether targeting DYRK1B could enhance the clinical efficacy of the PARP inhibitor Niraparib and expand its clinical indications, offering a promising combination therapeutic strategy for precise and effective clinical treatment of HGSOC.

## 2. Materials and Methods

### 2.1. Data Sources and Acquisition

The *DYRK1B* mRNA expression data of pan-cancer were obtained from The Cancer Genome Atlas (TCGA) database through the bioinformatics website (https://www.bioinformatics.com.cn, accessed on 5 May 2025). The RNA-Seq expression profiles of ovarian cancer were downloaded from the UCSC Xena platform. Four GEO datasets were downloaded (Table S1). The expression patterns of the DYRK1B protein in normal and cancerous ovarian tissues were analyzed using the HPA database (https://www.proteinatlas.org/, accessed on 18 May 2025).

### 2.2. Cell Culture and Main Reagents

The HGSOC cell lines, namely OVCAR4 (RRID: CVCL_1627), SNU119 (RRID: CVCL_5014), and COV362 (RRID: CVCL_2420), were generously provided by Prof. Hailing Cheng from the Central Laboratory of the Second Affiliated Hospital of Dalian Medical University. All human cell lines have been authenticated using STR profiling within the last three years. And all experiments were performed with mycoplasma-free cells. Among them, the COV362 cell line, originally isolated from the pleural effusion of a patient with endometrioid ovarian cancer, has been subsequently characterized by genomic and transcriptomic analyses as a model for HGSOC harboring a pathogenic BRCA1 mutation and is widely used as an in vitro model for BRCA-mutant HGSOC [23,24,25,26,27]. All cell lines were cultured in RPMI 1640 medium supplemented with 10% fetal bovine serum and 1% penicillin–streptomycin. The cultures were maintained in a humidified incubator with a 5% CO_2_ atmosphere at 37 °C. The main reagents and their sources are shown in Table S2.

### 2.3. Patients and Samples

From June 2024 to March 2025, we collected fresh tumor tissue samples and ascites from patients with HGSOC who underwent surgical treatment or ultrasound-guided abdominal puncture. A total of 15 patients were included in the study (Table S3). All operations were in compliance with the ethical guidelines approved by the Ethics Committee of the First Affiliated Hospital of Dalian Medical University (Ethics Number: PJ-KS-KY-2023-474), and the written informed consent of all participants or their legal guardians was obtained.

### 2.4. Screening and Analysis of Differential Genes

Differential gene expression analysis was performed using the DESeq2 package. (version 1.48.1) The identification criteria for the differentially expressed genes (DEGs) are: |log2 fold change (Log2 FC)| > 1, false detection rate (FDR) < 0.05 (corrected by Benjamini–Hochberg). The DEGs were analyzed using the STRING database to construct a protein–protein interaction (PPI) network, and the Hub genes were identified using the MCODE and CytoNCA plugins in Cytoscape (version 3.10.3).

Gene Ontology (GO) and Kyoto Encyclopedia of Genes and Genomes (KEGG) pathway enrichment analysis used clusterProfiler and GOplot packages to functionally annotate the identified genes. Gene Set Enrichment Analysis (GSEA) was performed on the ovarian cancer dataset GSE32062. Gene Set Variation Analysis (GSVA) was performed on GSE140082 to calculate enrichment scores for DNA repair-related gene sets from the Molecular Signatures Database (MSigDB). Significantly enriched gene sets were required to meet all of the following criteria: (1) *p* < 0.05; (2) FDR < 25%; (3) |Normalized Enrichment Score (NES)| > 1.0.

### 2.5. Analysis of Immune Cell Infiltration

Based on the immune cell characteristic gene sets in the MSigDB, the ssGSEA algorithm in the GSVA package (version 2.4.4) was used to calculate the relative infiltration levels of 24 types of immune cells.

### 2.6. Kaplan–Meier Survival Analysis

The prognostic significance of *DYRK1B* mRNA expression in ovarian cancer and ovarian serous carcinoma was evaluated using the Kaplan–Meier Plotter database (https://kmplot.com/analysis/, accessed on 18 May 2025). Patients were stratified into high-/low-expression groups using platform-optimized cutoff values. Subgroup analyses were performed based on clinical stage, histological grade, TP53 mutation status, surgical approach, and chemotherapy regimen.

### 2.7. Cell Viability Assay (MTT) and Drug Combination Analysis

Cells were seeded in 96-well plates at 3000 cells per well (100 µL of a 3 × 10^4^ cells/mL suspension). Following 72 h of treatment, 20 μL of MTT solution (5 mg/mL) was added to each well. After 4 h, DMSO was added to each well to dissolve the formazan crystals. Absorbance was measured at 490 nm using the Multimode Microplate Reader (Tecan Infinite M200, Tecan Group Ltd., Männedorf, Switzerland). Drug interactions were evaluated using the Chou–Talalay combination index (CI) method [28]. CI values were calculated by CompuSyn software (version 1.0.1, ComboSyn, Inc., Paramus, NJ, USA).

### 2.8. Flow Cytometric Apoptosis Analysis

Cells were seeded in 6-well plates at 75,000 cells per well (2.5 mL of a 3 × 10^4^ cells/mL suspension) and treated with drugs for 72 h. Apoptosis was detected using the Annexin V-FITC/PI double staining assay. Samples were analyzed by a BD FACSCanto II flow cytometer (BD Biosciences, San Jose, CA, USA).

### 2.9. Western Blot Analysis

Equal amounts of protein were separated by SDS-PAGE and transferred to PVDF membranes. The membranes were blocked with 5% skim milk, followed by incubation with primary antibodies and HRP-conjugated secondary antibodies. Protein bands were visualized using ECL reagent, and grayscale value was detected with a chemiluminescence imaging system (ChemiScope 6100, Clinx Science Instruments Co., Ltd., Shanghai, China). Detailed information on all primary antibodies used in this study, including the supplier, catalog number, and dilution ratio, is provided in Table S4.

### 2.10. Organoid Culture and Drug Sensitivity Analysis

The culture methods for organoids were performed as outlined in [29]. The detailed composition of the organoid culture medium is provided in Table S5. The organoids were re-added to the 96-well plates (10 μL/well). After treatment, its viability was assessed by MTT.

### 2.11. Statistical Analysis

All statistical analyses and data visualization were performed using R software (version 3.6.3, R Foundation for Statistical Computing, Vienna, Austria) and GraphPad Prism 10.0 (GraphPad Software, San Diego, CA, USA). Student’s *t*-test was applied to normally distributed data, while the Wilcoxon rank-sum test was used for non-parametric data. Data are presented as mean ± standard deviation (SD) from biological replicates. Multiple group comparisons were analyzed using one-way analysis of variance (ANOVA). Statistical significance levels (*p* values): * *p* < 0.05; ** *p* < 0.01; *** *p* < 0.001; **** *p* < 0.0001.

## 3. Results

### 3.1. DYRK1B Overexpression in Human Ovarian Cancer Correlates with Poor Prognosis

**Figure 1:**
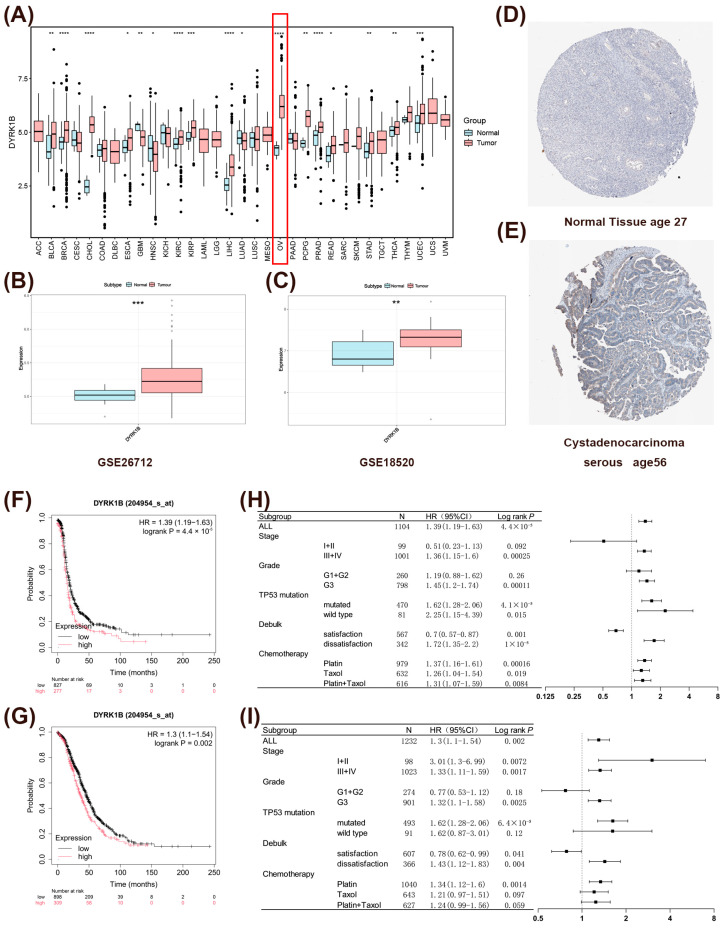
Correlation analysis of high expression of DYRK1B and prognosis of ovarian cancer. (**A**) Differences in *DYRK1B* expression between different malignant tumors and normal tissues in TCGA data. The red box highlights the differential expression of *DYRK1B* in ovarian cancer. * *p* < 0.05; ** *p* < 0.01; *** *p* < 0.001; **** *p* < 0.0001. (**B**,**C**) Expression differences in *DYRK1B* in ovarian cancer tissues and normal tissues from the GSE26712 (**B**) and GSE18520 (**C**) datasets. (**D**,**E**) Immunohistochemical staining of DYRK1B protein in normal fallopian tube tissues (**D**) and ovarian tissues from serous cystadenocarcinoma (**E**). (**F**–**I**) Stratified PFS curves (**F**), OS survival curves (**G**) and subgroup analysis of prognostic factors related to PFS (**H**) and OS (**I**) based on the high and low expression of *DYRK1B* mRNA in serous ovarian cancer of patients. Forest plots display hazard ratios (HRs) with 95% confidence intervals (CIs). (The log-rank test, Log rank *p* < 0.05 as considered statistically significant, protective factor: HR < 1, risk factor: HR > 1.)

To systematically investigate the tissue-specific expression patterns of *DYRK1B* across multiple cancer types, differential expression analysis using pan-cancer datasets from TCGA revealed that *DYRK1B* mRNA was significantly highly expressed in 14 human malignant tumors, including ovarian cancer, compared with the corresponding normal tissues (Figure 1A). Further, the differential expression analysis on the GSE26712 and GSE18520 ovarian cancer datasets also confirmed that the expression of *DYRK1B* mRNA was significantly upregulated in ovarian cancer tissues (Figure 1B,C). Additionally, through the HPA online database, we found that DYRK1B protein expression was not detected in normal ovarian tissues (Figure 1D), while it showed overexpression in serous cystadenocarcinoma tissues (Figure 1E). Subsequently, the prognostic analysis indicated that while no significant differences in PFS or OS were observed between *DYRK1B* high- and low-expression groups in the overall ovarian cancer cohort (Figure S1), patients with serous ovarian cancer in the high *DYRK1B* expression group showed significantly shorter PFS and OS (Figure 1F,G). Additionally, subgroup analysis indicated that *DYRK1B* could serve as a biomarker for poor prognosis in ovarian serous carcinoma, especially in patients with advanced stage, high grade, TP53 mutation, and those who did not achieve maximal cytoreductive surgery (R0 resection) (Figure 1H,I). In conclusion, the expression of DYRK1B is significantly upregulated in ovarian cancer and is closely related to the poor prognosis of ovarian serous carcinoma, suggesting that it may become a potential therapeutic target for ovarian cancer.

### 3.2. Functional Analysis of DYRK1B in Ovarian Cancer

**Figure 2:**
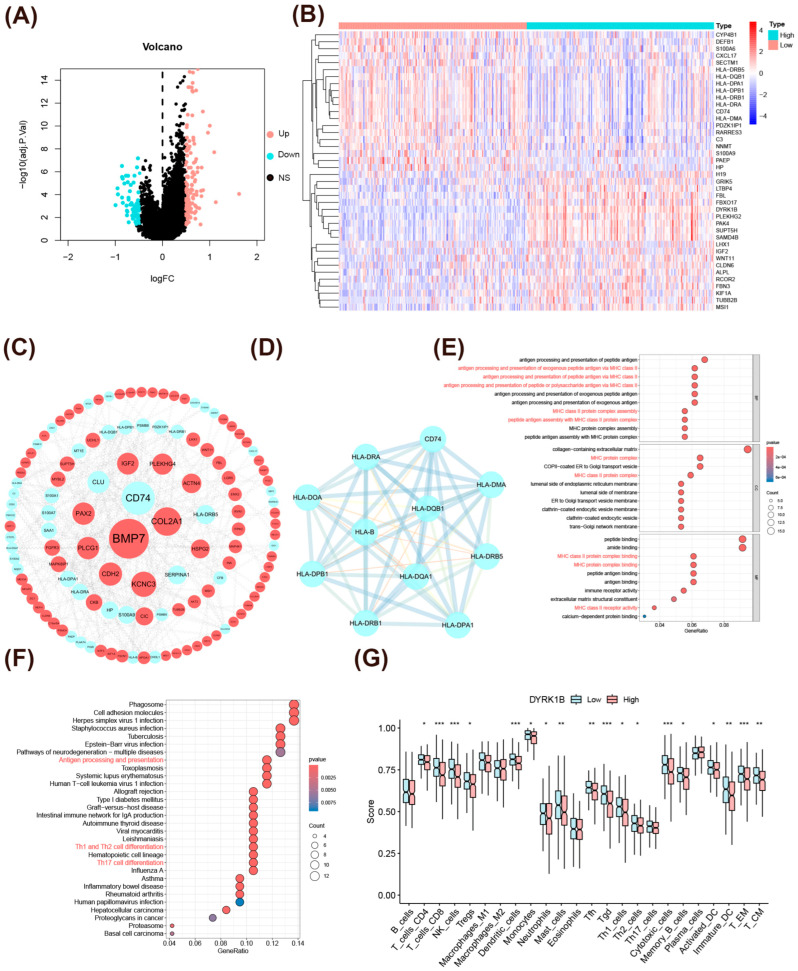
Single-gene differential analysis and immune infiltration analysis of DYRK1B. (**A**) Single-gene difference analysis of *DYRK1B* in a volcanic map. (**B**) A cluster heat map observes the clustering relationship of significantly DEGs in the *DYRK1B*-high-expression group (red) versus the *DYRK1B*-low-expression group (blue). The top 40 DEGs are ranked in ascending order of the adjusted *p*-value. (**C**) PPI network of DEGs. (**D**) HUB gene interaction network. (**E**) The biological processes that DEGs may be involved in. The horizontal axis represents the enrichment ratio (gene ratio), and the vertical axis represents the significantly enriched cellular components (CCs), molecular functions (MFs), and biological processes (BPs). (**F**) The signaling pathways that DEGs may be involved in. (**G**) Analysis of the intergroup differences in the infiltration of 24 immune cells between the low-expression and high-expression groups of *DYRK1B* (Wilcoxon test). * *p* < 0.05; ** *p* < 0.01; *** *p* < 0.001.

To gain deeper insights into the biological function of DYRK1B in ovarian cancer, we compared the transcriptome differences between ovarian cancer samples with high and low *DYRK1B* expression. The DEGs analysis showed that a total of 195 significantly differentially expressed genes were identified, among which 136 genes were upregulated, and 59 genes were downregulated (Figure 2A,B). Based on these DEGs, a PPI network was constructed (Figure 2C). In this network, the spatial position of a gene node reflects its connectivity: nodes located closer to the center indicate stronger interconnectivity with other genes, suggesting their potential role as hubs in signal transduction [30]. Furthermore, 11 hub genes negatively correlated with DYRK1B expression were identified through the MCODE plugin (Figure 2D). These genes are related to major histocompatibility complex (MHC) class II molecules [31,32]. Afterward, GO analysis revealed that the biological processes were primarily enriched in peptide antigen and MHC protein complex assembly, as well as antigen processing and presentation by MHC class II molecules (Figure 2E). KEGG pathway analysis revealed significant enrichment in antigen processing and presentation, along with immune pathways involved in effector T cell subset differentiation (Figure 2F). For further validation, we found that the infiltration levels of 18 types of immune cells in the *DYRK1B*-low-expression group were significantly higher than those in the high-expression group (Figure 2G). Using nine different deconvolution algorithms from the TIMER database, we systematically evaluated the correlation between *DYRK1B* expression and the abundance of tumor-infiltrating immune cells, further supporting the above conclusions (Figure S2). These outcomes indicate that DYRK1B may contribute to immune evasion in ovarian cancer by suppressing immune cell infiltration, thereby promoting tumor progression.

### 3.3. Correlation of DYRK1B Overexpression with Ferroptosis, ROS Metabolism, and DNA Repair in Ovarian Cancer

**Figure 3:**
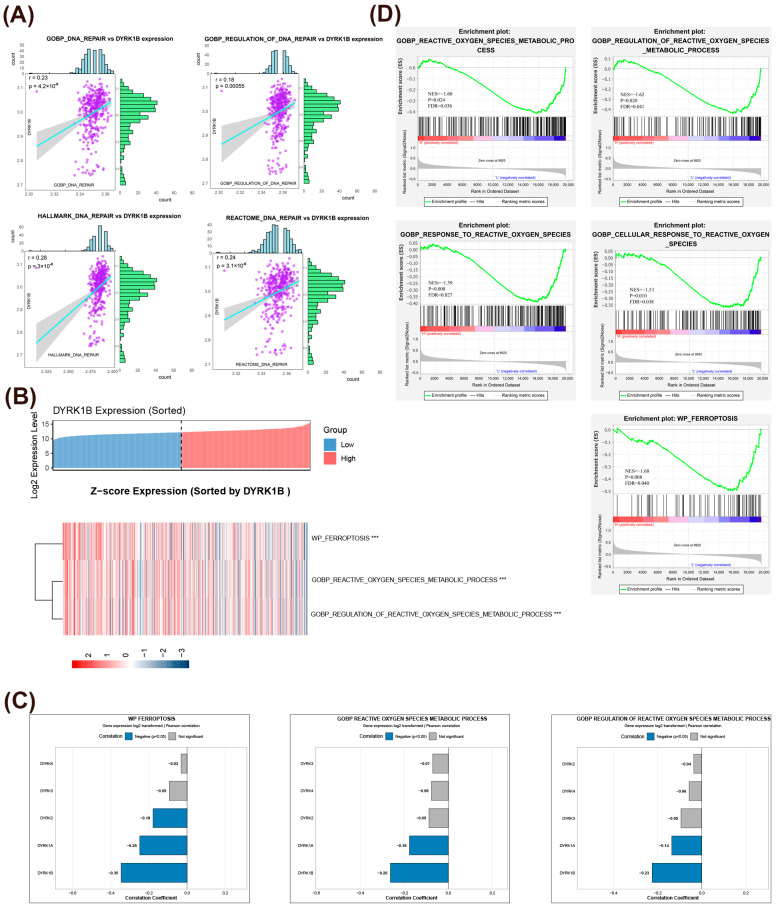
Functional enrichment analysis based on the TCGA database and the GEO database. (**A**) Correlation analysis was conducted on the HALLMARK or GO characteristic scores of the biological processes related to DNA repair of *DYRK1B* using the GSE140082 ovarian cancer dataset (Pearson’s test). (**B**) Correlation analysis of *DYRK1B* expression level with ferroptosis and ROS pathway in TCGA ovarian cancer data: the upper part shows the heat map of *DYRK1B* gene expression level, and the samples are sorted from low to high expression level. The lower part shows the Z-score expression heatmap of the sets related to ferroptosis genes, with colors ranging from blue to red indicating the Z-score from low to high. *** *p* < 0.001. (**C**) Each subgraph corresponds to a biological process and its correlation coefficient with the expression of DYRK family genes and their significance. (**D**) The enrichment map of HALLMARK or GO characteristic pathways related to *DYRK1B* expression using the ovarian cancer dataset GSE32062.

The GSVA and GSEA analyses were performed to investigate *DYRK1B* downstream target networks in ovarian cancer. The GSVA assessment of the DNA repair signature score in the GSE140082 dataset revealed a significant positive correlation between the expression level of the *DYRK1B* gene and the expression of DNA repair-related genes and their related regulatory pathways (Figure 3A). It is suggested that DYRK1B may promote the survival of ovarian cancer cells by facilitating the mechanism of DNA damage repair.

Functional enrichment analysis of TCGA ovarian cancer datasets demonstrated that *DYRK1B* exhibited the strongest association with ROS regulation and ferroptosis pathways among all DYRK family members, with its expression levels displaying significant negative correlations with these critical biological processes (Figure 3B,C). Interestingly, these related patterns are consistent with the enrichment analysis results in the independent ovarian cancer dataset (GSE32062) (Figure 3D). All the above findings indicate that DYRK1B may promote the survival of ovarian cancer cells and generate chemotherapy-period resistance through mechanisms such as promoting DNA damage repair, inhibiting ferroptosis, and regulating ROS-related pathways.

### 3.4. Synergistic Anti-Proliferative Effects of Combined AZ191 and Niraparib Treatment in HGSOC Cells

**Figure 4:**
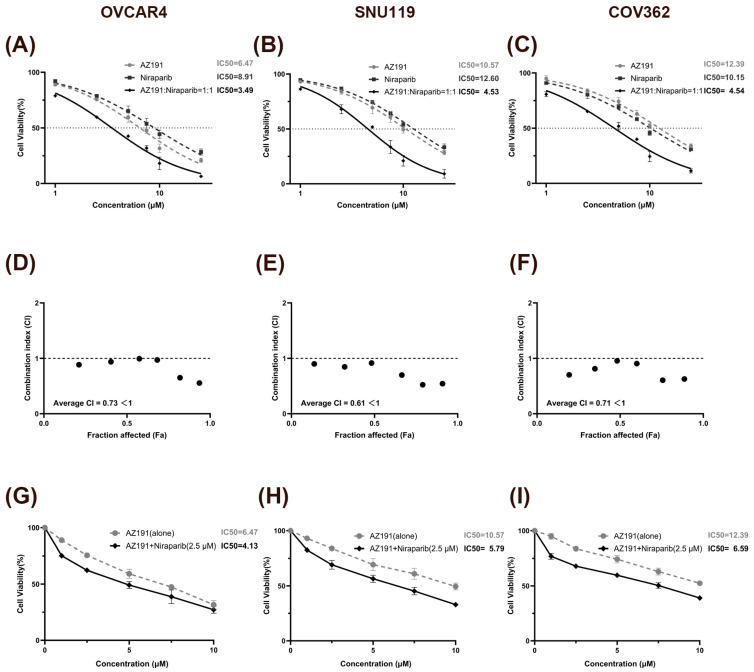
Synergistic inhibitory effects of AZ191 combined with Niraparib on HGSOC cell proliferation. (**A**–**C**) Dose–response curves of AZ191 and Niraparib as single agents or in 1:1 combination after 72 h of treatment in three HGSOC cell lines (**A**: OVCAR4, **B**: SNU119, **C**: COV362). (**D**–**F**) CI values in three HGSOC cell lines. FA, fraction affected. Each black dot indicates the CI value corresponding to a given Fa level for the 1:1 fixed-ratio combination of AZ191 and niraparib in the three HGSOC cell lines. (**G**–**I**) Dose–response curves and IC_50_ values of AZ191 alone and AZ191 combined with Niraparib (2.5 μM) after 72 h of treatment in three HGSOC cell lines.

To determine whether AZ191 enhances HGSOC sensitivity to Niraparib, our investigation revealed that the combination treatment significantly reduced IC50 values compared to Niraparib monotherapy across three HGSOC cell lines (Figure 4A–C). In addition, synergy analysis demonstrated that all specified drug combinations exhibited synergistic effects (CI < 1) (Figure 4D–F). Notably, targeting DYRK1B increases the sensitivity to Niraparib in both BRCA-mutation COV362 and BRCA wild-type OVCAR4 or SNU119 cell lines, suggesting that even for the maintenance treatment of HGSOC with intact HRR function, DYRK1B may also serve as a combined target for enhancing the efficacy of Niraparib.

Although PARP inhibitors generally exhibit lower toxicity profiles compared to conventional chemotherapeutics, a substantial proportion of patients still require dose reduction or treatment discontinuation due to hematological adverse events, including anemia and thrombocytopenia. Given these clinical constraints, dose optimization for PARP inhibitor combination strategies represents a critical challenge warranting immediate investigation. To address this concern, we employed a fixed low-dose Niraparib concentration (2.5 μM) in combination with gradient concentrations of AZ191. The concentration was selected based on the IC20 values calculated from the dose–response curves of each cell line to Niraparib (OVCAR4: 2.29 μM; SNU119: 3.03 μM; COV362: 2.55 μM), with an approximate value of 2.5 μM chosen as the fixed concentration. The experimental evidence showed that combination treatment resulted in significantly reduced IC50 values compared to monotherapy (Figure 4G–I). Therefore, in the subsequent experiments, Niraparib was used at the above-mentioned specified concentration (2.5 μM).

### 3.5. Combined AZ191 and Niraparib Treatment Synergistically Induces Apoptosis, DNA Damage, and Ferroptosis in HGSOC Cells

**Figure 5:**
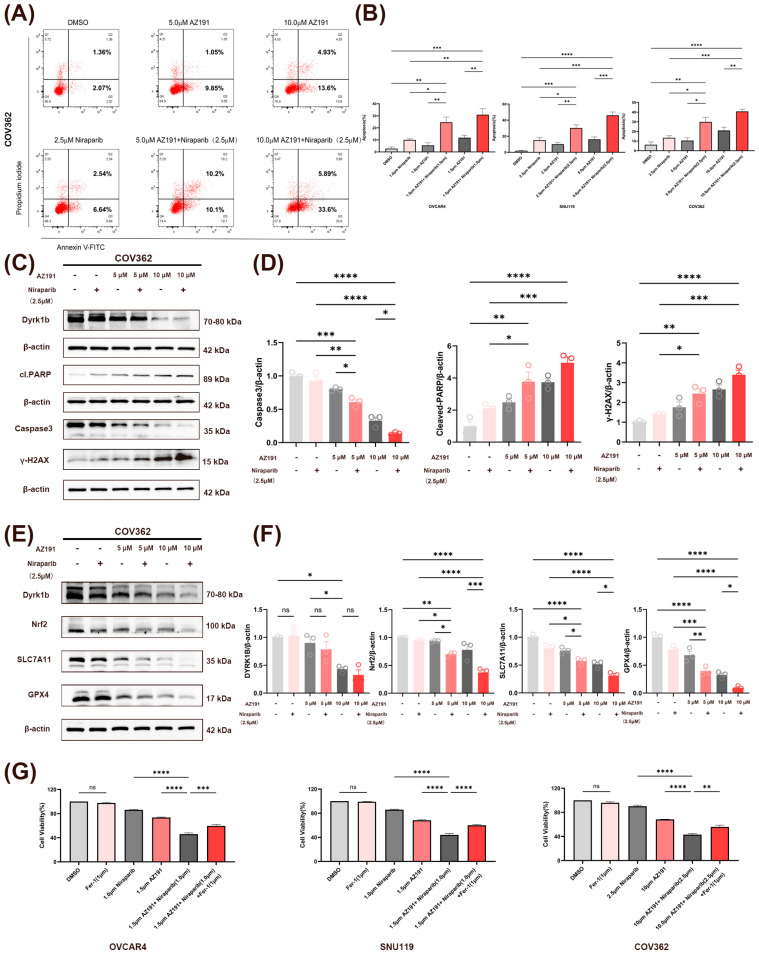
Effects of AZ191 and Niraparib alone or in combination on apoptosis and ferroptosis in HGSOC cells. (**A**,**B**) Apoptosis rates of three HGSOC cell lines after 72 h of treatment with AZ191 and Niraparib (SNU119 and COV362: 2.5 μM; OVCAR4: 1.0 μM) alone or in combination, as analyzed by Annexin V-FITC/PI double-staining flow cytometry, *n* = 3. (**A**) Representative plots for COV362. (**B**) Quantification of apoptosis rates for all three lines. (In preliminary experiments, treatment of OVCAR4 cells with 2.5 μM Niraparib for 72 h induced a high level of late apoptosis and cell debris, which interfered with the accurate quantification of early apoptotic events. Therefore, the drug concentration for flow cytometry analysis was adjusted to 1 μM to ensure the quality of the experimental data.) (**C**,**D**) Western blot analysis of protein levels of DYRK1B, Cleaved PARP, Caspase 3, and γH2AX in COV362 cell lines after 72 h of treatment with AZ191 and Niraparib (2.5 μM) alone or in combination, *n* = 3. (**E**,**F**) Western blot analysis of protein levels of Nrf2, SLC7A11, and GPX4 in COV362 cell lines after 72 h of treatment; β-actin was used as an internal reference, *n* = 3. (**G**) Cell viability was assessed by MTT assay, three cell lines were treated with AZ191 and Niraparib alone or in combination± Fer-1for 72 h, *n* = 3. * *p* < 0.05; ** *p* < 0.01; *** *p* < 0.001; **** *p* < 0.0001; *ns* indicates no statistical significance.

Our investigation of the synergistic effects of AZ191 and Niraparib revealed that the combination therapy significantly increased the apoptosis rate across all three HGSOC cell lines compared to vehicle control and monotherapy groups. Moreover, apoptotic rates in the combination groups exhibited a dose-dependent increase in response to AZ191. (Figure 5A,B and Figure S3A). Further Western blot analysis in COV362 cells with high DYRK1B expression demonstrated that AZ191 dose-dependently downregulated DYRK1B expression. Furthermore, the combination groups significantly activated both apoptotic and DNA damage pathways, as evidenced by the marked downregulation of Caspase3 and upregulation of Cleaved PARP and γH2AX, compared to the control and monotherapy groups. (Figure 5C,D). The trends of changes in apoptosis and DNA damage-related proteins in SNU119 and OVCAR4 cell lines under treatment with AZ191 and Niraparib alone and in combination were consistent with those observed in COV362 (Figure S3B).

Previous studies have shown that the Nrf2/SLC7A11/GPX4 signaling axis plays a pivotal role in ferroptosis defense through coordinated regulation of cystine uptake, GSH synthesis, and lipid peroxide clearance [33,34]. Hence, this study found through Western blot analysis that the combined use of AZ191 and Niraparib significantly downregulated the expression of the three proteins mentioned above more than either one alone (Figure 5E,F). Importantly, addition of the ferroptosis inhibitor Ferrostatin-1 (1 μM) partially reversed this cytotoxic effect of the combination group, indicating the involvement of ferroptosis in the synergistic cell death (Figure 5G).

Together, the above experiments confirmed that AZ191 can synergistically intensify the DSB accumulation, apoptosis and ferroptosis induced by Niraparib in HGSOC cells.

### 3.6. Combined AZ191 and Niraparib Treatment Synergistically Demonstrates Anti-Tumor Activity in HGSOC Organoids

**Figure 6:**
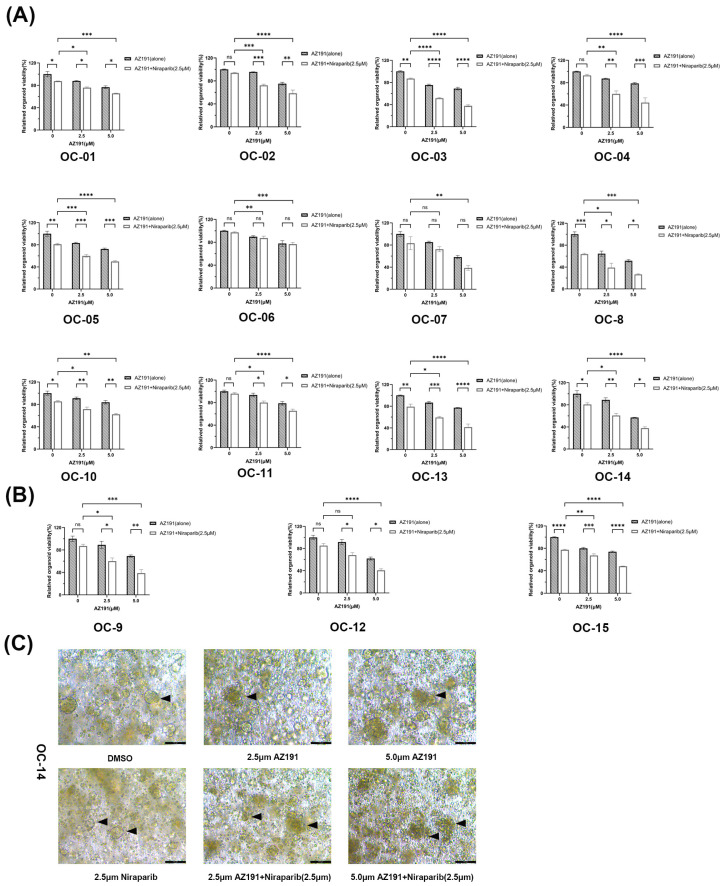
The synergistic inhibitory effect of AZ191 combined with Niraparib on the proliferation of HGSOC tissues and ascites organoids. (**A**,**B**) Evaluation of drug efficacy in patient-derived HGSOC tissues (**A**) and ascites (**B**) organoids. The absorbance of each group was detected by the MTT method, and the relative survival rate of cells was calculated, *n* = 3. (**C**) After AZ191 and Niraparib (2.5 μM) were used as monotherapy or in combination with one HGSOC tissue-derived organoid for 72 h, the formation of organoids in each group was observed. (The black arrows represent the comparison of morphological changes in HGSOC tissue-derived organoids in each group.) Scale bar, 100 μm. *p* < 0.05; ** *p* < 0.01; *** *p* < 0.001; **** *p* < 0.0001; *ns* indicates no statistical significance.

To verify the clinical translational potential of the AZ191–Niraparib combination therapy, we successfully established organoid models derived from tumor specimens (12 lines) and malignant ascites samples (3 lines) of patients with primary HGSOC. These models maintained stable growth kinetics characteristics consistent with the original tumor structure during the culture process (Figure S4). The results of the combined action of AZ191 and Niraparib on HGSOC organoids showed that, in 10 tumor tissue-derived organoids (10/12, 83.33%) and 3 ascites cell-derived organoids (3/3, 100%), the combined treatment had a significant inhibitory effect compared with the control group and the monotherapy group (Figure 6A,B). Surprisingly, the survival rate of the organoids in the combined group of AZ191 and Niraparib was significantly lower compared to the Niraparib monotherapy group; moreover, regarding the inhibitory effect of the combined treatment, there was no obvious difference between the HRD and HRP group. Morphological analysis revealed that the combined treatment significantly disrupted the structural integrity of the organoids, as evidenced by: (i) reduced organoid formation and individual organoid volume; (ii) blurred organoid edges and decreased intercellular adhesion; (iii) structural disintegration of the organoids, accompanied by fragmentation and loss of their original glandular morphology (Figure 6C). The HGSOC patient-derived organoid model shows a high response rate to the combined treatment of AZ191 and Niraparib, providing a new strategy for the precise and effective clinical treatment of ovarian cancer and expanding the clinical indications of the PARP inhibitor Niraparib.

## 4. Discussion

Currently, the limited beneficiary group of PARP inhibitors, the reduction and discontinuation of drug dosage in some patients due to adverse reactions, and acquired drug resistance, among other challenges, highlight the urgent need for in-depth optimization of PARP inhibitor treatment strategies [6]. Herein, we reported that the expression of *DYRK1B* mRNA and protein is upregulated in ovarian cancer and can mediate multiple mechanisms leading to a poor prognosis of ovarian serous carcinoma through bioinformatics. Our data further demonstrated that COV362 and SNU119 cells, which exhibit relatively high DYRK1B expression (Figure S5), displayed strong synergistic effects, with mean combination index (CI) values of 0.71 and 0.61, respectively. In contrast, OVCAR4 cells, characterized by the lowest DYRK1B expression among the three lines, showed a markedly weaker synergistic effect (mean CI = 0.73). These findings further support DYRK1B expression as a key determinant of response to this combination therapy. Further targeted inhibition of DYRK1B with a specific inhibitor, AZ191, enhanced the sensitivity of HGSOC cell lines and organoids to Niraparib. This significant effect was also observed even in four organoids with HRP and either the OVCAR4 or SNU119 cell line with no BRCA1/2 mutation, indicating that DYRK1B is an attractive potential target for enhancing the efficacy of PARP inhibitors in the maintenance treatment of HGSOC, regardless of homologous recombination status.

It has been reported [35] that PARP inhibitors mainly induce transcription–replication conflicts leading to DNA damage that requires the homologous recombination (HR) pathway for repair, which causes “synthetic lethality” in tumor cells with HRD. Additionally, genetic inactivation or pharmacological inhibition of DYRK1B impairs DSB-induced gene silencing, compromising DNA repair pathways [14,15], which is closely related to the drug sensitivity of PARP inhibitors. We also proposed for the first time that AZ191 combined with Niraparib can synergistically induce DSB accumulation and cell apoptosis in tumor cells.

Beyond DNA damage repair pathways, the present study also revealed that highly expressed *DYRK1B* can negatively regulate ferroptosis and ROS levels. Among the ferroptosis signaling regulatory pathways, GPX4 is an important target for studying the induction of ferroptosis in cancer cells. Nrf2, as a stress transcription factor, can increase the expression of SLC7A11 and GPX4. Activating the Nrf2/SLC7A11/GPX4 signaling pathway exerts antioxidant effects and can inhibit ferroptosis [33,34,36]. Therefore, through cell experiments, this study indicated that combined dual-target inhibition may synergically exacerbate iron death in tumor cells by inhibiting the Nrf2/SLC7A11/GPX4 signaling pathway. There are also studies [20] on the combination of PARP inhibitors, which showed that PARP inhibitors combined with GPX4 inhibitors lead to a significant increase in DNA damage and eventually lead to the death of cancer cells with an intact HR pathway. This coincides with the direction of our findings, and together provides new treatment options for HR-sensitive patients. Nevertheless, the evidence for ferroptosis in this study is only suggestive, based on reduced protein levels and partial rescue by ferrostatin-1. Future studies using direct readouts (e.g., lipid ROS, GSH depletion, iron content and MDA) are required for further validation.

It is worth noting that we discovered that the expression of DYRK1B is significantly negatively correlated with multiple MHC II class molecule-related proteins. It is known that the main function of MHC II class molecules is to present exogenous antigens in the form of antigen peptide–MHC II class molecule complex to T cells, leading to the activation and differentiation of T cells. Their expression and functional abnormalities are closely related to tumor immune evasion and prognosis [31]. Previous studies have demonstrated that in ovarian cancer, the expression of MHC class II pathway proteins is associated with prolonged patient survival [32]. This suggests that DYRK1B may promote the initiation and progression of ovarian cancer and impair patient prognosis by suppressing the expression of MHC class II pathway proteins. Therefore, whether DYRK1B inhibition affects the tumor immune microenvironment through the regulation of MHC-II in HGSOC cells remains an important question to be addressed in our future research. In addition, we found that higher *DYRK1B* expression was associated with reduced immune cell infiltration. Similar to this result, a study [37] on pancreatic ductal adenocarcinoma revealed that gene deletion or drug inhibition of DYRK1B could significantly recruit tumor-killing macrophages, and simultaneously downregulate the phagocytic checkpoint signaling molecules CD24 (the “don’t eat me” signal) on the surface of cancer cells, thereby enhancing the phagocytic clearance of tumor cells. However, further studies in ovarian cancer are required to determine whether targeting DYRK1B can improve the efficacy of immunotherapy.

However, we acknowledge certain limitations in this study. First, although AZ191 has been extensively validated as a selective DYRK1B inhibitor with minimal off-target effects at the concentrations used [38,39], and siRNA-/shRNA-mediated *DYRK1B* knockdown has been shown to phenocopy AZ191 treatment [40,41], future mechanistic studies should further incorporate *DYRK1B* knockdown models to definitively rule out potential off-target effects. Second, the MTT assay [42,43,44,45] combined with bright-field imaging to document morphological changes—the approach for organoid viability assessment used in this study—has inherent limitations in accurately capturing responses in three-dimensional cultures; future studies should adopt more accurate and suitable methods, such as CellTiter-Glo^®^ 3D combined with immunofluorescence staining and confocal imaging. Finally, although our in vitro and organoid data point to a potential therapeutic benefit of combining AZ191 with Niraparib, the translational significance of this combination remains preliminary due to the lack of toxicity data on normal ovarian epithelial cells, as well as in vivo efficacy and safety evidence. Additional validation using animal models and normal cell selectivity assays is therefore necessary before clinical applicability can be considered.

## 5. Conclusions

In summary, this study demonstrated that targeting DYRK1B can mediate DSB accumulation to promote apoptosis and downregulate the Nrf2/SLC7A11/GPX4 signaling pathway to exacerbate ferroptosis, thereby increasing the sensitivity of HGSOC cells to the PARP inhibitor Niraparib. In addition, the HGSOC patient-derived organoid established in this study verified the efficacy of the combined treatment of AZ191 and Niraparib, providing preliminary evidence that may inform future individualized treatment strategies and further investigation of combination therapies with PARP inhibitors.

## Data Availability

Data can be made available upon request.

## Supplementary Materials

The following supporting information can be downloaded at: https://www.mdpi.com/article/10.3390/biomedicines14040939/s1. Figure S1: Kaplan–Meier evaluation of the relationship between *DYRK1B* expression and the survival of patients with ovarian cancer. Figure S2: Correlation landscape between *DYRK1B* expression and tumor-infiltrating immune cells in ovarian cancer. Figure S3: Effects of AZ191 and Niraparib alone or in combination on apoptosis and DNA damage in HGSOC cells. Figure S4: Construction of human organoids for high-grade serous ovarian cancer. Figure S5: The expression of DYRK1B in five high-grade serous ovarian cancer cell lines. Table S1: GEO Dataset. Table S2: Main reagents and the sources. Table S3: Information on patients for organoids. Table S4: List of antibodies used in this study. Table S5: Components of the organoid culture medium. Reference [46] is cited in the Supplementary Materials.

## Abbreviations

The following abbreviations are used in this manuscript:

DSB	Double-strand break
DYRK1B	Dual-specificity tyrosine phosphorylation-regulated kinase 1B
GPX4	Glutathione peroxidase 4
HRD	Homologous recombination deficiency
HRP	Homologous recombination proficiency
HGSOC	High-grade serous ovarian cancer
NRF2	Nuclear factor erythroid 2-related factor 2
OS	Overall survival
PARP	Poly (ADP-ribose) polymerase
PFS	Progression-free survival
ROS	Reactive oxygen species
SLC7A11	Solute carrier family 7 member 11
