# Supplementary material for: DYRK1B Inhibition by AZ191 Sensitizes High-Grade Serous Ovarian Cancer to Niraparib Through Promoting Apoptosis and Ferroptosis
Source: Biomedicines. 2026 Apr 20;14(4):939. doi: 10.3390/biomedicines14040939 (PMC13114077; doi:10.3390/biomedicines14040939)
Supplement: Supplementary file 1 [file biomedicines-14-00939-s001.zip › Figure S1.pdf]

Figure S1.:

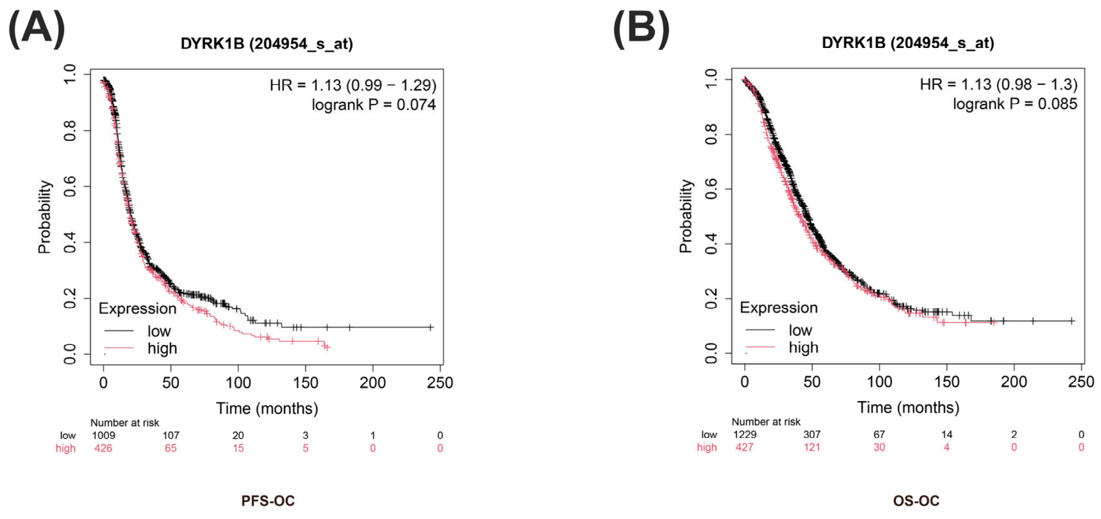

Figure S1: Kaplan-Meier evaluation of the relationship between *DYRK1B* expression and the survival of patients with ovarian cancer

（A-B）Stratified PFS survival curves (A) and OS survival curves (B) based on the high and low expression of *DYRK1B* mRNA in serous ovarian cancer of patients.
