# Supplementary material for: DYRK1B Inhibition by AZ191 Sensitizes High-Grade Serous Ovarian Cancer to Niraparib Through Promoting Apoptosis and Ferroptosis
Source: Biomedicines. 2026 Apr 20;14(4):939. doi: 10.3390/biomedicines14040939 (PMC13114077; doi:10.3390/biomedicines14040939)
Supplement: Supplementary file 1 [file biomedicines-14-00939-s001.zip › Figure S2.pdf]

Figure S2.:

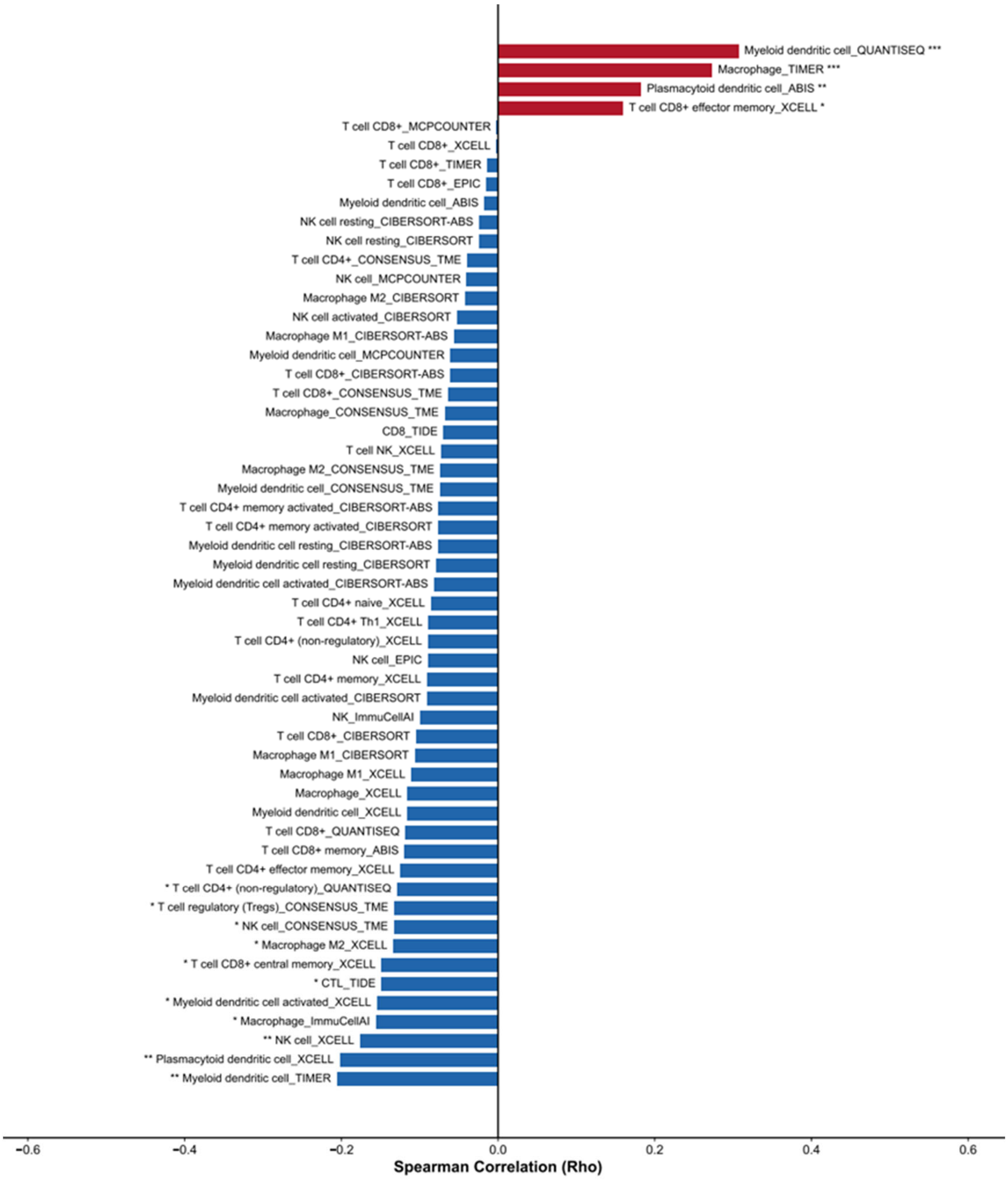

Figure S2: Correlation landscape between *DYRK1B* expression and tumor-infiltrating immune cells in ovarian cancer.

Spearman rank correlation analysis was performed to evaluate the association between *DYRK1B* expression levels and the abundance of diverse immune cell subsets in ovarian cancer (OV) cohorts (n = 422). Multiple well-established immune deconvolution algorithms (including CIBERSORT, xCell, QUANTISEQ, ImmuCellAI, TIMER, MCP-counter, EPIC, ABIS, and TIDE) were applied to estimate immune infiltration levels. The length of the horizontal bars represents the Spearman correlation coefficient (Rho). Red bars pointing to the right indicate positive correlations (Rho > 0), whereas blue bars pointing to the left indicate negative correlations (Rho < 0). （* adj. $p < 0.05$, ** adj. $p < 0.01$, *** adj. $p < 0.001$.）
