# Supplementary material for: DYRK1B Inhibition by AZ191 Sensitizes High-Grade Serous Ovarian Cancer to Niraparib Through Promoting Apoptosis and Ferroptosis
Source: Biomedicines. 2026 Apr 20;14(4):939. doi: 10.3390/biomedicines14040939 (PMC13114077; doi:10.3390/biomedicines14040939)
Supplement: Supplementary file 1 [file biomedicines-14-00939-s001.zip › Figure S3.pdf]

Figure S3.:

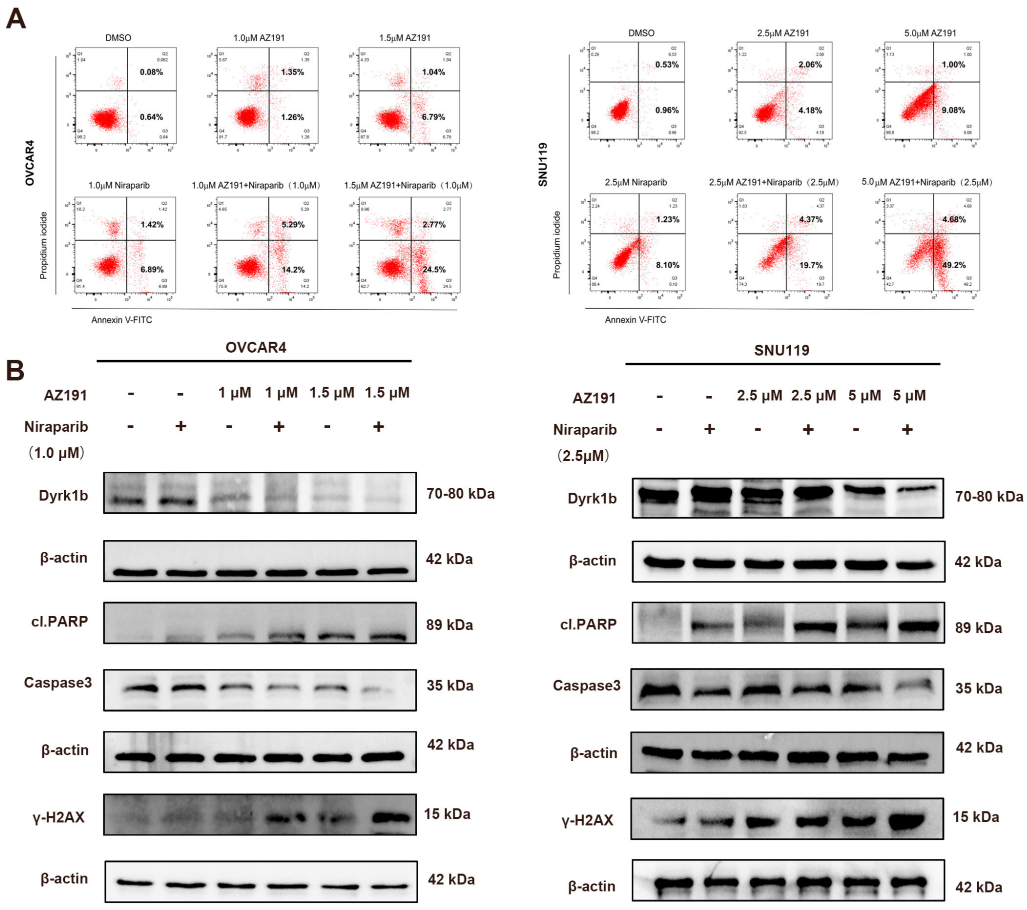

Figure S3: Effects of AZ191 and Niraparib alone or in combination on apoptosis and DNA damage in HGSOC cells .

(A) Representative flow cytometry plots of Annexin V‑FITC/PI double staining for OVCAR4 and SNU119 cells after 72 h treatment with AZ191 and Niraparib alone or in combination (SNU119: 2.5 μM; OVCAR4: 1.0 μM). The representative plots for COV362 are shown in Figure 5A.
(B) Representative Western blot images of apoptosis‑related proteins (cleaved PARP, cleaved caspase‑3) and DNA damage‑related protein (γH2AX) in SNU119 and OVCAR4 cells under the same treatment conditions. These changes were consistent with those observed in COV362 (see Figure 5 in the main text).
