# Supplementary material for: DYRK1B Inhibition by AZ191 Sensitizes High-Grade Serous Ovarian Cancer to Niraparib Through Promoting Apoptosis and Ferroptosis
Source: Biomedicines. 2026 Apr 20;14(4):939. doi: 10.3390/biomedicines14040939 (PMC13114077; doi:10.3390/biomedicines14040939)
Supplement: Supplementary file 1 [file biomedicines-14-00939-s001.zip › Figure S4.pdf]

Figure S4.:

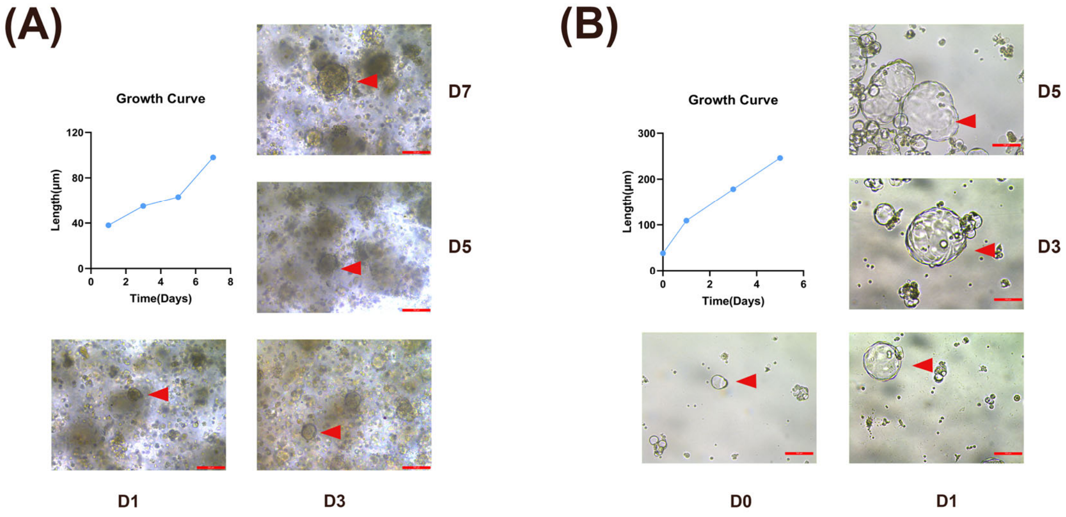

Figure S4.: Construction of human organoids for high-grade serous ovarian cancer

(A) A construction example of an organoid from a patient-derived HGSOC tissue and its growth curve;(B) A construction example of ascites organoids for HGSOC from a patient and its growth curve; HGSOC： high-grade serous ovarian cancer(The red arrow points to the organoid being tracked.)
