# Supplementary material for: DYRK1B Inhibition by AZ191 Sensitizes High-Grade Serous Ovarian Cancer to Niraparib Through Promoting Apoptosis and Ferroptosis
Source: Biomedicines. 2026 Apr 20;14(4):939. doi: 10.3390/biomedicines14040939 (PMC13114077; doi:10.3390/biomedicines14040939)
Supplement: Supplementary file 1 [file biomedicines-14-00939-s001.zip › Table S1.pdf]

Table S1：

Table S1　GEO Dataset

**Dataset**	**Sample number (Tumor/Control)**	**Tumor group**	**Control group**	**Platform**
GSE26712	185/10	HGSOC	OSE	GPL96
GSE18520	53/10	HGSOC	OSE	GPL570
GSE32062	10/ 0	HGSOC	/	GPL570
GSE140082	380/ 0	OC	/	GPL14951

Abbreviations: HGSOC：high-grade serous ovarian cancer；OC：ovarian cancer；OSE：ovarian surface epithelium； GPL： GEO platform.
