# Supplementary material for: DYRK1B Inhibition by AZ191 Sensitizes High-Grade Serous Ovarian Cancer to Niraparib Through Promoting Apoptosis and Ferroptosis
Source: Biomedicines. 2026 Apr 20;14(4):939. doi: 10.3390/biomedicines14040939 (PMC13114077; doi:10.3390/biomedicines14040939)
Supplement: Supplementary file 1 [file biomedicines-14-00939-s001.zip › Table S2.pdf]

**Table S2：**

**Table S2 Main reagents and the sources**

| Ragent | Catalog Number | Source |
|---|---|---|
| Niraparib | HY-10619 | MedChem Express |
| Hydrocortisone | HY-N0583 | MedChem Express |
| MTT | S6821 | Selleck |
| Nicotinamide | S1899 | Selleck |
| AZ191 | S7338 | Selleck |
| Advanced DMEM/F12 | 12634028 | Thermo Fisher |
| Hepes | 15630080 | Thermo Fisher |
| B27 | 17504044 | Thermo Fisher |
| L-Glutamine 100x | A4000225301 | Thermo Fisher |
| Primocin | ANT-PM-05 | InvivoGen |
| Clostridium histolyticum collagenase | C9407 | Sigma |
| N - Acetylcysteine | 38520-57-9 | Sigma |
| Forskolin | 66575-29- 9 | Sigma |
| β - Estradiol | 50-28-2 | Sigma |
| A83 - 01 | 909910-43-6 | Sigma |
| SB202190 | 152121-30-7 | Sigma |
| Heregulinβ - 1 | H7660 | Sigma |
| HGF | 100 - 39 | Peprotech. |

| | | |
|---|---|---|
| Human EGF | AF-100-15 | Peprotech. |
| Human FGF10 | 100-26 | Peprotech. |
| Y27632 | 129830 -38- 2 | Abmole Bioscience |
| Dyrk1B | 2703S | Cell Signaling Technology |
| Caspase 3 | 9662S | Cell Signaling Technology |
| cleaved - PARP | 9541S | Cell Signaling Technology |
| NRF2 | 20733S | Cell Signaling Technology |
| SLCA711 | 1291S | Cell Signaling Technology |
| GPX4 | 52455S | Cell Signaling Technology |
| β-actin | 20536-1-AP | Proteintech Group. |
| the HRP - conjugated Goat Anti - Rabbit IgG (H + L) | SA00001-2 | Proteintech Group. |
| Annexin V-FITC/PI double - staining apoptosis detection kit | KGA1102 | KeyGEN BioTECH |

Abbreviations:NRF2: Nuclear factor erythroid 2-related factor 2; SLCA711: Solute Carrier Family 7 Member 11; GPX4: Glutathione Peroxidase 4.
