# Supplementary material for: DYRK1B Inhibition by AZ191 Sensitizes High-Grade Serous Ovarian Cancer to Niraparib Through Promoting Apoptosis and Ferroptosis
Source: Biomedicines. 2026 Apr 20;14(4):939. doi: 10.3390/biomedicines14040939 (PMC13114077; doi:10.3390/biomedicines14040939)
Supplement: Supplementary file 1 [file biomedicines-14-00939-s001.zip › Table S3.pdf]

Table S3：

Table S3 Information on patient for organoids

Number	Tissue /Ascites Source	Genetic Testing Results
OC-1	HGSOC Tumor	HRD（+）
OC-2	HGSOC Tumor	HRD（+）
OC-3	HGSOC Tumor	-
OC-4	HGSOC Tumor	HRD（-）
OC-5	HGSOC Tumor	-
OC-6	HGSOC Tumor	HRD（-）
OC-7	HGSOC Tumor	HRD（+）
OC-8	HGSOC Tumor	HRD（+）
OC-9	HGSOC Ascites	-
OC-10	HGSOC Tumor	HRD（+）
OC-11	HGSOC Tumor	HRD（-）
OC-12	HGSOC Ascites	HRD（-）
OC-13	HGSOC Tumor	-
OC-14	HGSOC Tumor	HRD（+）
OC-15	HGSOC Ascites	-

Note: HGSOC, high-grade serous carcinoma; HRD, homologous recombination deficiency; "-", genetic testing was not performed for the patient. HRD status is evaluated based on the presence of pathogenic variants of BRCA1/2 combined with the genomic instability score (GIS), samples not meeting these criteria were classified as HRP [1].

[1] Patient Assessment and Therapy Planning Based on Homologous Recombination Repair Deficiency. Genomics Proteomics Bioinformatics. Volume 21, Issue 5, October 2023, Pages 962–975, https://doi.org/10.1016/j.gpb.2023.02.004
