# Supplementary material for: DYRK1B Inhibition by AZ191 Sensitizes High-Grade Serous Ovarian Cancer to Niraparib Through Promoting Apoptosis and Ferroptosis
Source: Biomedicines. 2026 Apr 20;14(4):939. doi: 10.3390/biomedicines14040939 (PMC13114077; doi:10.3390/biomedicines14040939)
Supplement: Supplementary file 1 [file biomedicines-14-00939-s001.zip › Table S4.pdf]

Table S4：

Table S4　List of antibodies used in this study

Antibody	Supplier	Catalog No.	Dilution
Primary Antibodies			
DYRK1B (D40D1) Rabbit mAb	Cell Signaling Technology	2703	1:1000
NRF2 (D1Z9C) XP® Rabbit mAb	Cell Signaling Technology	12721	1:1000
xCT/SLC7A11 (D2M7A) Rabbit mAb	Cell Signaling Technology	12691	1:1000
GPX4 Antibody	Cell Signaling Technology	52455	1:1000
Cleaved PARP (Asp214) Antibody	Cell Signaling Technology	9541	1:1000
γ-H2AX (Ser139) Antibody	Cell Signaling Technology	2577	1:1000
H2AX Antibody	Cell Signaling Technology	7631	1:1000
β-Actin (13E5) Rabbit mAb	Cell Signaling Technology	4970	1:1000
Secondary Antibodies			
HRP-conjugated Goat Anti-Rabbit IgG	Proteintech (Sanying, Wuhan)	SA00001-2	1:5000
