# Supplementary material for: DYRK1B Inhibition by AZ191 Sensitizes High-Grade Serous Ovarian Cancer to Niraparib Through Promoting Apoptosis and Ferroptosis
Source: Biomedicines. 2026 Apr 20;14(4):939. doi: 10.3390/biomedicines14040939 (PMC13114077; doi:10.3390/biomedicines14040939)
Supplement: Supplementary file 1 [file biomedicines-14-00939-s001.zip › Table S5.pdf]

Table S5：

Table S5 Components of the organoid culture medium

Reagent / Component	Manufacturer / Supplier	Catalog Number
Advanced DMEM/F-12 (1X)	Thermo Fisher Scientific, USA	12634010
HEPES (1 M)	Thermo Fisher Scientific, USA	15630080
B-27 Supplement (50X)	Thermo Fisher Scientific, USA	17504044
L-Glutamine (100X)	Thermo Fisher Scientific, USA	25030081
Nicotinamide	Selleck Chemicals, USA	S4255
N-Acetylcysteine	Sigma-Aldrich, USA	A9165
Forskolin	Sigma-Aldrich, USA	F6886
β-Estradiol	Sigma-Aldrich, USA	E2758
A83-01	Sigma-Aldrich, USA	SML0788
SB202190	Sigma-Aldrich, USA	S7067
Y-27632	AbMole Bioscience, USA	M1817
Recombinant Human HGF	PeproTech, USA	100-39

Reagent / Component	Manufacturer / Supplier	Catalog Number
Recombinant Human Heregulin β-1	PeproTech, USA	100-03
Recombinant Human EGF	PeproTech, USA	100-15
Recombinant Human FGF10	PeproTech, USA	100-26
