# Supplementary figures and images for: DYRK1B Inhibition by AZ191 Sensitizes High-Grade Serous Ovarian Cancer to Niraparib Through Promoting Apoptosis and Ferroptosis
Source: Biomedicines. 2026 Apr 20;14(4):939. doi: 10.3390/biomedicines14040939 (PMC13114077; doi:10.3390/biomedicines14040939)

Figure S5.:

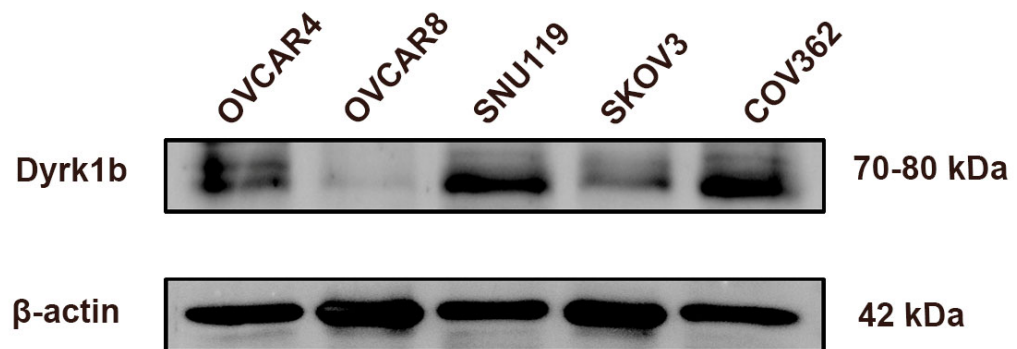

Figure S5.: The expression of DYRK1B in five high-grade serous ovarian cancer cell lines.

Supplement: Supplementary file 1 [file biomedicines-14-00939-s001.zip › Figure S5.pdf]
